# When Free Is Not for Me: Confronting the Barriers to Use of Free Quitline Telephone Counseling for Tobacco Dependence

**DOI:** 10.3390/ijerph13010015

**Published:** 2015-12-22

**Authors:** Christine Sheffer, Sharon Brackman, Charnette Lercara, Naomi Cottoms, Mary Olson, Luana Panissidi, Jami Pittman, Helen Stayna

**Affiliations:** 1 Sophie Davis School of Biomedical Education, City College of New York, New York, NY 10031, USA; charnettelercara@gmail.com (C.L.); lpanissidi@med.cuny.edu (L.P.); jpittman@med.cuny.edu (J.P.); hstayna@med.cuny.edu (H.S.); 2 Fay W Boozman College of Public Health, University of Arkansas for Medical Sciences, Little Rock, AR 72205, USA; csheffer@med.cuny.edu; 3 Walnut Street Works, Inc., Helena-West Helena, AR 72342, USA; ncottoms@aol.com (N.C.); molson144@yahoo.com (M.O.)

**Keywords:** quitline, tobacco dependence, barriers to treatment, low-income, ethnic minority, rural

## Abstract

Remarkable disparities in smoking rates in the United States contribute significantly to socioeconomic and minority health disparities. Access to treatment for tobacco use can help address these disparities, but quitlines, our most ubiquitous treatment resource, reach just 1%–2% of smokers. We used community-based participatory methods to develop a survey instrument to assess barriers to use of the quitline in the Arkansas Mississippi delta. Barriers were quitline specific and barriers to cessation more broadly. Over one-third (34.9%) of respondents (*n* = 799) did not have access to a telephone that they could use for the quitline. Respondents reported low levels of knowledge about the quitline, quitting, and trust in tobacco treatment programs as well as considerable ambivalence about quitting including significant concerns about getting sick if they quit and strong faith-based beliefs about quitting. These findings suggest quitlines are not accessible to all lower socioeconomic groups and that significant barriers to use include barriers to cessation. These findings suggest targets for providing accessible tobacco use treatment services and addressing concerns about cessation among lower income, ethnic minority, and rural groups.

## 1. Introduction

Tobacco use remains the leading cause of preventable death and disease in the United States (U.S.) and is now a leading contributor to health disparities [1,2,3,4,5,6]. In the US, the prevalence of cigarette smoking has declined significantly; however, remarkable tobacco use disparities have emerged among groups defined by income, ethnic minority status, geographic region, and other characteristics resulting in disproportionate burdens of tobacco-related disease [7]. For example, the smoking prevalence for those with annual household incomes less than $15,000 is 34%, three times that of those with incomes greater than $49,999 (11.9%) [8]. Significantly more rural residents live in poverty than residents of metropolitan areas [9] and more African Americans live in poverty than whites [10] suggesting that urbanicity and race are important factors to consider when examining tobacco disparities.

Comprehensive tobacco control programs aim to reduce tobacco use by providing all tobacco users with effective, evidence-based treatment for tobacco dependence [11]. Proactive telephone counseling delivered through “quitlines”, is widely available in the US, United Kingdom, Canada, and elsewhere, and is intended to increase the accessibility of high-quality treatment for tobacco dependence [12,13] Evidence suggests that quitlines attract lower income, African American, and rural smokers [14]; however, quitlines only appear to reach at most 1%–2% of smokers [12,15].

Arkansas is a largely rural state with a high proportion of African American and lower income residents, and significant tobacco use disparities [8,16]. Several Arkansas counties lie in the heart of the Mississippi Delta region, a predominately lower-income, underserved area with a large proportion of African American residents and some of the lowest health status indicators in the United States [3,16,17,18,19]. At the time this study was conducted, the Arkansas quitline had been providing evidence-based treatment for tobacco dependence free of charge for several years; however, the Arkansas Mississippi Delta counties demonstrated rates of participation 1–2 standard deviations below the mean smoker participation rate for other counties in the state [20]. Recent evidence suggests that a lack of consistent telephonic services along with skepticism about the treatment provided through quitlines and an aversion to communicating by telephone contribute to non-use of quitlines among HIV-positive and methadone maintained smokers [21,22].

This study is the second phase of a project in which we initiated a formative inquiry to use of the Arkansas quitline in the Arkansas Mississippi delta region. In the first phase we used a community-based participatory approach to conduct a qualitative examination of the barriers to using the quitline [23]. Barriers identified in the qualitative study included those specific to using the quitline including a lack of knowledge about and trust in the quitline as well as limited availability of appropriate telephonic resources [23]; however, the focus groups in this study also identified barriers to cessation more broadly as barriers to use of the quitline including a lack of motivation to quit related to disadvantaged socioeconomic context and concerns about the negative health effects of quitting. The qualitative findings also suggested that the population in this region hold strong beliefs about the role of faith in the process of quitting that affect decisions to use the quitline and decisions to quit. While the findings from the qualitative study provide considerable insight and are quite compelling, they cannot be generalized beyond the individuals who participated in the group discussions. Moreover, the qualitative findings provide no quantification in terms of relative intensity and frequency and no differentiation can be made between demographic groups.

In this, the second phase of this project, we used a community-based participatory approach to quantify the findings from the qualitative study. We worked with our community partners to develop a survey instrument and administer this instrument in the two counties in which we conducted the qualitative study. We approached this as an exploratory, descriptive inquiry with the specific aim of quantifying selected findings from the qualitative study. We expected the barriers identified in qualitative study to be present, but there was little evidence to predict the degree to which the barriers existed in this population.

## 2. Experimental Section

### Methods

**Instrument development**. Using community-based participatory principles [24], the research team members (*i.e.*, Christine Sheffer, Sharon Brackman, Naomi Cottoms, and Mary Olson) prioritized the qualitative findings from phase one of the project for examination in phase two. The final survey items reflect a consensus preference among team members to assess barriers to the quitline specifically and barriers to cessation more broadly. Items included questions about trust in tobacco cessation programs, concerns about using telephone counseling in general, and the availability of telephonic resources as well as levels of motivation, confidence, and knowledge about quitting; concerns about getting sick when one quits; and the strength of beliefs in the role of God in deciding to quit and quitting. Community partners (Naomi Cottoms and Mary Olson) recommended that we limit the size of the survey to one-page and administer demographic questions after the content items to increase respondent participation. We pilot tested the instrument with *n* = 10 community members recruited from downtown Helena West-Helena, AR. Feedback was used to revise the items for clarity. 

**Population surveyed**. We administered the survey in the same counties in which phase one, the qualitative study, was conducted: Cross (pop. 19,237) and Lee (pop. 11,545) counties. The median household income in Cross County was $30,287 and in Lee County $21,795. The percent of persons living below poverty level was 19.6% in Cross County and 29.1% in Lee County. African American residents comprise 23.6% of Cross County and 57.0% of Lee County [16,25].

**Survey instrument**. The survey instrument included seven demographic items (age, sex/gender, race/ethnicity, income, employment status, county of residence); tobacco use status (current, former, never used regularly); type of tobacco used; and 16 items assessing knowledge, attitudes, and beliefs about tobacco use, cessation, the quitline, and tobacco treatment program services. Knowledge, attitudes, and belief items used a 0–10 discrete analogue scale (0 = none at all, 10 = most possible) for responses. There was one open-ended question about what would help respondents quit tobacco the most; and one item asking whether respondents had access to a telephone to talk privately and conveniently about quitting tobacco for a total of 27 items.

**Procedure**. This study was approved by the institutional review boards of the University of Arkansas for Medical Sciences and City College of New York. Specially trained community members approached individuals attending local businesses and community events and asked them to complete the survey. No individuals under 16 years of age were asked to complete the survey. Blank surveys were handed to participants on a clipboard. All individuals 16 years or older who were willing to complete the survey were included. Participation was voluntary, anonymous, and without remuneration. Data was collected in March and April of 2009.

**Data preparation**. Preliminary analyses called for combining specific categories for tobacco use status, income, and race. Some respondents (*n* = 217) did not circle a response to “Tobacco use? Current user, former user, never used regularly”. Of these respondents *n* = 180 circled one or more tobacco products used: “Cigarettes, cigars, chew, dip, snuff, or other” indicating that they used tobacco products, either presently or in the past; and *n* = 37 did not circle a tobacco product and were eliminated from the analysis because we could find no evidence of tobacco use status. Other respondents (*n* = 86) identified as former tobacco users, but also reported using tobacco products. In order to address missing tobacco use status items as well as the ambiguity of actual tobacco use among a subset of former tobacco users, we created two tobacco use status categories of which we had strong confidence were distinct: (1) We combined respondents who reported current tobacco use, former tobacco use, and any tobacco use into one current/former tobacco user group; (2) We maintained the never users without change. For the purposes of this study, this decision is supported by the fact that potential users of the quitline will include both former tobacco and current tobacco users. Former tobacco users are at high risk of relapse. In fact, 95% of tobacco users who quit relapse in the first year [26]. These two categories also accommodate tobacco users who are unwilling to identify as such. Of note, in a recent study, 60.2% of non-daily smokers identified as non-smokers [27].

In order to balance cell sizes for significance testing, the six income levels were collapsed into three categories of roughly equal size. Respondents who identified as other than Black/African American or White (*n* = 14) were eliminated from significance testing for racial differences because the cell sizes were too small for comparison purposes.

**Data analysis**. Data were analyzed using SPSS version 20. Descriptive analyses were conducted (means, standard deviations, and frequencies) followed by significance testing (analysis of variance and χ^2^ analyses) examining differences between current, former, and never tobacco users; and differences by income level and race (α = 0.05). Standardized residuals (R = {observed-expected}/square root of expected) were employed to identify sources of significant differences (standardized residual > 2.00) for categorical variables [28]. The Bonferroni post-hoc test was used for multiple comparisons [29].

The responses (*n* = 527) to the open-ended questions were analyzed using an inductive, grounded approach [30]. Members of the research team independently reviewed the responses and generated a list of emergent categories or themes. A theme was defined as a group of responses with the same meaning as indicated by repetition or replication of meaning. The team members then met, compared, and analyzed their results, agreed on a final list of categories, and coded the original list. The final coding comprehensively categorized all responses. Chi-square analyses were used to examine differences in the frequencies of response categories by sex/gender, race, and income level.

## 3. Results and Discussion

### 3.1. Results

Respondents (*n* = 799) were primarily middle-aged, of lower income, and residents of Cross (49.4%), Lee (39.2%), and Arkansas (6.0%) counties. The majority of respondents were employed full (48.0%) or part-time (18.2%). Over 90% of respondents reported annual household incomes of less than $35,000. The majority (84.8%) of respondents reported current or former tobacco use, 15.2% reported never using tobacco. Two-thirds of respondents self-identified as Black or African American. Current/former tobacco users tended to be older, were more likely to be male, and were less likely to report annual household incomes of $35,000 or greater than never tobacco users. See Table 1.

**Table 1:** Demographic characteristics of respondents.

Characteristic		Total Mean (SD)/% (*n*)	Tobacco Use	
			Current/Former	Never
			(*n* = 646)	(*n* = 116)
			Mean (SD)/% (*n*)	Mean (SD)/% (*n*)
Male (%) *		47.1 (346)	48.2 (287)	36.9 (41)
Mean age in years *		42.7 (15.5)	43.3 (15.5)	39.7 (15.23)
Race (%)	White	30.8 (230)	29.4 (179)	36.3 (41)
	Black/African American	67.3 (503)	68.4 (416)	62.8 (71)
	Native American	0.4 (3)	0.5 (3)	0 (0)
	Multi-ethnic	1.5 (11)	1.6 (10)	0.9 (1)
Annual Household income (%) **	<$10,000	26.4 (186)	27.6 (161)	16.2 (17)
	$10–14,999	25.7 (181)	26.8 (156)	21.0 (22)
	$15–24,999	24.7 (174)	25.6 (149)	22.9 (24)
	$25–34,999	12.2 (86)	11.3 (66)	16.2 (17)
	$35–49,999 **	7.1 (50)	6.2 (36)	13.3 (14)
	>$50,000 **	4.0 (28)	2.6 (15)	10.5 (11)
Employment Status (%)	Full-time	48.0 (356)	47.4 (290)	53.7 (58)
	Part-time	18.2 (135)	18.3 (112)	17.6 (19)
	Homemaker	5.7 (42)	5.6 (34)	6.5 (7)
	Disabled	11.5 (85)	11.8 (72)	8.3 (9)
	Unemployed	10.1 (75)	10.6 (65)	5.6 (6)
	Retired	6.6 (49)	6.4 (39)	8.3 (9)

Differences examined between current/former and never tobacco users. * *p* < 0.05; ** *p* < 0.01.

The quitline-specific barriers among current/former tobacco users included a remarkable proportion of respondents (34.9%) who did not have access to a telephone to talk privately and conveniently about quitting tobacco. They also reported relatively low levels of knowledge about the quitline and moderate to low levels of trust in programs to help them quit both within and outside their communities. The cessation-related factors included moderate levels of desire to quit, confidence in their ability to quit, perceived importance of quitting, and perceived knowledge about quitting tobacco. Respondents also endorsed faith-based beliefs at moderately strong levels including the belief that God would give them the power to quit when it is the right time to quit and that prayer and trust in God are the best way to quit tobacco. Nonetheless, the pattern of some of the cessation-related factors reflected considerable ambivalence. The level of importance attributed to reasons for quitting was within one standard deviation of the level of importance attributed to reasons for not quitting. Respondents expressed concern that they would get sick or get cancer if they quit tobacco, as well as concern that they would get sick if they did not quit tobacco. See Table 2.

**Table 2:** Resources, knowledge, attitudes, and beliefs associated with quitting tobacco and using the quitline among current/former tobacco users.

Survey Items ^†^	Percent (*n*) or Mean (SD)
Percent without telephone services (*n*)	34.9 (197)
Confidence to quit for good	6.01 (3.43)
Importance of quitting	6.10 (3.34)
Motivation to quit	5.82 (3.34)
Knowledge about quitting	5.18 (3.24)
Knowledge about the quitline	3.14 (3.2)
Trust in programs outside your community to help quit	4.19 (3.08)
Trust in programs inside your community to help quit	4.67 (2.83)
Concern that something bad will happen to you if you use a statewide program to quit	3.47 (3.03)
Concern about giving out personal information over the telephone to someone who is helping you to quit	5.12 (3.32)
Importance of reasons for not quitting tobacco	4.29 (3.07)
Importance of reasons for quitting tobacco	6.05 (3.17)
Concern that you will get sick if you quit tobacco	4.01 (3.39)
Concern that you will get cancer if you quit	3.86 (3.39)
Concern about getting sick if you do not quit	4.99 (3.84)
Belief that God will give you the power to quit when it is the right time	6.62 (3.43)
Belief that prayer and trust in God is the best way to quit	6.68 (3.32)

**^†^** Items assessed on a discrete analogue scale of 0–10, where 0 = not at all and 10 = the most possible.

There were more differences by income level than by race. See Table 3 and Table 4. The highest income group (≥$35,000) reported the greatest levels of importance and knowledge about quitting, but the lowest level of knowledge about the quitline. The lowest income group endorsed the greatest levels of importance to reasons for not quitting and the lowest levels of importance to reasons for quitting. The lowest income group also expressed the greatest concern about getting sick and getting cancer if they quit as well as the greatest concern that something bad would happen to them if they used a statewide program to quit. The lowest income group was also more concerned about giving out personal information over the telephone than the middle income group. African American or Black respondents were more confident that they could quit tobacco for good, reported a greater level of knowledge about the quitline, and lower levels of trust in programs to help them quit outside their communities than White respondents.

A total of *n* = 527 respondents completed the open-ended question: *n* = 16 of these indicated that they had already quit and were eliminated; *n* = 27 of these provided multiple responses or a compound response such that the response could be categorized into two categories, leaving *n* = 511 relevant respondents with *n* = 538 responses. The frequency of response categories are reflected in Table 5. While the frequencies of response categories by sex/gender (χ^2^ = 22.36, df 12, *p* = 0.03) and income level (χ^2^ = 36.3, df 24, *p* = 0.05) reached significance, none of the standardized residuals within categories reached an absolute value of 2 and thus no comparisons were reported. No difference was found in the frequencies of response categories by race (χ^2^ = 16.1, df 12, *p* = 0.20).

**Table 3:** The significant differences among responses between current/former tobacco users with different income levels.

Survey Items ^†^	≤$14,999 (*n* = 313)	$15,000–34,999 (*n* = 212)	≥$35,000 (*n* = 50)
Importance of quitting	5.7 (3.8) **^a^**	6.4 (3.2) **^b^**	7.9 (2.8)**^a,b^**
Knowledge about quitting	4.8 (3.3) **^a^**	5.4 (3.2)	6.1 (2.9) **^a^**
Knowledge about the quitline	3.4 (3.3) **^a^**	2.8 (2.9)	1.9 (2.7) **^a^**
Importance of reasons for not quitting	4.6 (3.1) **^a^**	3.9 (2.8) **^a^**	3.9 (3.7)
Importance of reasons for quitting	5.7 (3.3) **^a,b^**	6.4 (2.9) **^a^**	7.4 (3.0) **^b^**
Concern about getting sick if you quit	4.5 (3.5) **^a,b^**	3.5 (3.0) ^a^	2.9 (3.6) **^b^**
Concern about getting cancer if you quit	4.3 (3.5) **^a,b^**	3.4 (3.1) **^a^**	3.0 (3.3) **^b^**
Concern that something bad will happen to you if you use a statewide program to quit	3.9 (3.2) **^a,b^**	3.0 (2.7) **^a^**	2.6 (2.9) **^b^**
Concern about giving personal information over the telephone to someone who is helping you quit	5.4 (3.4) **^a^**	4.6 (3.3) **^a^**	5.8 (3.3)

**^†^** Items assessed on a discrete analogue scale of 0–10, where 0 = not at all and 10 = the most possible; Means with the same superscript (e.g., **^a^** or **^b^**) are significantly different.

**Table 4:** The significant differences among Black and White current/former tobacco users.

Survey Items ^†^	White	Black/African American
Confidence to quit for good?	5.4 (3.4)	6.0 (3.4)
Knowledge about the quitline	2.6 (3.1)	3.1 (3.1)
Trust in programs outside your community to help you quit	4.6 (2.8)	3.9 (3.1)

**^†^** Items assessed on a discrete analogue scale of 0–10, where 0 = not at all and 10 = the most possible.

**Table 5:** Frequency of responses to open-ended question: “What would help you quit tobacco the most?”.

Categories	Frequency of Response (*n*)
Knowledge, education, need more information about quitting, do not know enough about it	16.0% (86)
Support from God, faith, religion	12.1% (65)
Nothing, hopeless, fatalistic response	11.5% (62)
Treatments or programs for tobacco cessation	11.2% (60)
Increasing the cost, economic factors, financial impact	10.6% (57)
Specific strategies such as exercise, cold turkey, smoking fewer cigarettes	10.2% (55)
Willpower, motivation, self-determination, trust in self, self-confidence, belief in ability, wanting to, intention (or lack thereof)	8.9% (48)
External factors including better environment, making cigarettes unavailable by law	7.8% (42)
Support from family, friends, children, not being alone in quitting	4.3% (23)
Support (unspecified)	2.8% (15)
Sickness, health concerns, personal illness	2.8% (15)
Change in life circumstances or lifestyle outside of one’s control	1.9% (10)
Total	100% (538)

### 3.2. Discussion

This study identified barriers to use of the tobacco counseling quitline among a primarily lower income, ethnic minority sample of residents in the Arkansas Mississippi Delta region. The findings have implications for identifying and addressing barriers to use of quitlines in particular, but also use of other tobacco dependence treatment services and to tobacco cessation in general among lower income, ethnic minority, and rural groups, many of whom experience significant tobacco disparities. Among the most obvious quitline-specific findings is that over one-third of current/former tobacco users did not have access to a telephone to engage in telephone counseling. While these results do not explain why this was the case, we speculate that lower income individuals who live in rural areas with no public transportation must travel long distances by automobile to reach basic resources (e.g., shopping, employment, *etc.*) and are more likely to devote limited resources to transportation (e.g., maintaining a vehicle, fuel, *etc.*) than maintaining a private telephone with free or unlimited minutes. This interpretation is supported by qualitative findings in which participants indicated that many tobacco users in their community had cell service for which they paid by the minute and/or shared with others and could not easily be used to speak privately with a health care provider [23]. One must have free available minutes to be able to use the quitline for free telephone counseling. These findings are also supported by previous studies in which a large proportion of low-income HIV positive smokers [21] and methadone maintained smokers [22] reported telephone availability as a barrier to use of the quitline. While quitlines are often assumed to be attractive and accessible to lower income groups, a lack of the telephonic resources needed to use the service clearly prevents access. Lazev *et al.* successfully engaged a significant proportion of low income smokers by providing cell phones to enable their participation in the treatment provided by the quitline [21]. More research is needed into the telephonic resources available to groups with tobacco disparities. New or enhanced strategies are needed to deliver evidence-based treatment for tobacco dependence to groups without free minutes.

Knowledge about the quitline and about quitting tobacco in general also appear to be significant barriers to using the quitline. Few current/former tobacco users in this study appeared to have sufficient information about the quitline to actually use the quitline. Given the apparent ubiquity of quitline advertising at the time of this study, we initially considered this finding to be remarkable; however, in the context of having limited telephonic resources, individuals might have been exposed to the advertising but considered the service “not for me” and did thus not attend to the messaging. Similarly, respondents as a whole reported only moderate levels of knowledge about quitting with the lowest income groups reporting significantly lower levels of knowledge. The most frequent response to the open-ended question was a desire for more knowledge, information, or education about quitting. These findings are similar to the findings of Christiansen *et al.* (2012) in which about 20% of the lower income respondents did not know or were not sure whether counseling and medications helped to quit smoking and over 40% did not think counseling and medications help to quit smoking [31]. More research is needed into how to effectively deliver more detailed information about quitting, the quitting process, and the use of the quitline and other treatment options.

Current/former tobacco users in this study were almost as concerned about getting sick or cancer from quitting as they were concerned about getting sick from *not* quitting. The lowest income respondents reported the highest levels of concerns about getting sick if they quit. This finding is supported by qualitative findings in which participants indicated that they had noticed that “bad things—like cancer—show up when people quit” [23]. These concerns are likely to reflect respondents’ observations that some tobacco users get sick when they quit. It is not uncommon to attribute causality to two co-occurring events, but the accurate interpretation, of course, is that quitters get sick because they are suffering the health consequences of tobacco use. This concern is not unfamiliar to clinicians who provide treatment for tobacco dependence and a quick search of the internet reveals that this concern is fielded on a number of informal advice sites (e.g., answers.yahoo.com and ehow.com). Interestingly, health concerns were one of the least frequently reported categories of responses to the open-ended question, “What would help you quit tobacco the most?” These findings suggest that educational efforts focused on the health consequences of tobacco use might not have a large impact on this or similar groups who have concerns that quitting can cause illness. More research is needed into how to proactively address concerns about getting sick from quitting tobacco among groups with tobacco disparities.

Even though current/former tobacco users reported levels of desire to quit, confidence in the ability to quit, importance of quitting, and knowledge about quitting consistent with previous surveys of non-treatment seeking tobacco users [31,32,33], the levels of importance attributed to the reasons for quitting and *not* quitting were within one standard deviation of each other suggesting ambivalence. Moreover, the lowest income group reported lower levels of importance for quitting and reasons for quitting and the highest levels of importance for the reasons for *not* quitting. Although many, if not all, tobacco users struggle with balancing reasons for quitting and not quitting and this affects motivation to quit, the third most frequent response category in the open-ended question was one of hopelessness (e.g., nothing can help me) which can significantly undermine motivation and confidence and suggests a significant struggle with perhaps maintaining motivation and confidence to quit. This struggle might be more difficult or simply different for individuals with added life struggles such as lower income and ethnic minority groups. More research is needed into how to help groups with tobacco disparities to address motivation and confidence to quit in this and similar groups.

Low levels of trust and specific concerns about using programs were identified as potential barriers to using the quitline and tobacco dependence treatment services in general. Respondents reported relatively low levels of trust in programs, concerns that something bad would happen as a consequence of using a state program, and concerns about giving out personal information over the telephone. The lowest income respondents reported the highest level of concern that something bad would happen if they used a program. This lack of trust in treatments for tobacco dependence is similar to Christiansen *et al.* (2012) in which nearly 50% of lower income respondents indicated that medications to help quit can be more dangerous than smoking [31]. Nonetheless, findings from the open-ended question indicate that a meaningful proportion of respondents think that treatments or programs for tobacco cessation would help them quit and that specific strategies are helpful. Many of the strategies listed were commonly utilized in evidence-based treatment suggesting that if their concerns were addressed, respondents would be open to evidence-based treatment. More research is needed into methods to increase trust in and address specific concerns about using tobacco dependence treatment programs among groups with tobacco disparities.

Our findings indicate that faith-based beliefs are important to respondents when thinking about quitting and the best way to quit. Respondents reported relatively strong beliefs that God will give you the power to quit when it is the right time and that prayer and trust in God are the best way to quit tobacco. Support from God, faith, and religion was also the second most frequently reported category of response in the open-ended question. These findings are inconsistent with the findings of Karvinen *et al.* (2014) in which God Locus of Health Control was found to be unrelated to smoking and other health beliefs [34]; however, the Karvinen study respondents were demographically dissimilar to the respondents in this study in terms of urbanicity, socioeconomic status, and race. Our findings invite speculation about the role of faith-based beliefs in quitting and treatment. These beliefs might be a potential barrier or alternatively, a reinforcing factor. The belief that God will provide the power when the time is right to quit and/or the belief that prayer and trust in God are the best way to quit might support an external locus of control and suppress proactive problem-solving, treatment seeking, and use of the quitline. Conversely, if these faith-based beliefs are combined with proactive efforts to quit and/or treatment seeking, they might serve to reinforce cessation efforts. Incorporating faith-based elements into treatment or messaging about the quitline might enhance trust in the program. More research is needed into the role of faith-based beliefs and how faith-based beliefs can support efforts to quit.

The limitations of this study include the unanticipated ambiguity in tobacco use status categories and a convenience sample of individuals who attended local businesses and community events, and what appears to be a high proportion of current/former tobacco users in the sample. While the tobacco use status categories of current, former, or never might appear discrete to researchers and clinicians, they might not discriminate well among tobacco users in the community. The community partners reported that some respondents used different types of tobacco occasionally and did not identify as current tobacco users. This notion is supported by the prevalence data which indicates that about 5.9% of the population in Arkansas in 2009 smoked some days, but not every day, and that the prevalence of non-daily or intermittent smoking is growing [8,35]. Lower income and African Americans have been reported to be among the growing population of intermittent smokers [35]. These individuals might not identify as “smokers” or “tobacco users”. We attribute the high proportion of current and former tobacco users in the sample to the manner in which the data were collected. We asked about all types of tobacco use, not just cigarettes, and grouped the current and former tobacco users together. For smoking alone, the prevalence of daily, non-daily, and former use among lower income groups in Arkansas was about 60% in 2009 [8]. Additionally, individuals who use tobacco might have been more inclined to complete the survey than individuals who did not use tobacco because the items were more relevant to tobacco users. Nonetheless, we believe the findings are germane and important because no level of tobacco use is safe and all current and former tobacco users are potential users of tobacco dependence treatment services. These findings are also limited by a lack of specificity in the reasons why respondents did not have access to a telephone to use the quitline (e.g., lack of free minutes, no landline, no cell phone, lack of privacy, sharing a telephone, *etc.*).

## 4. Conclusions

These findings indicate that a large proportion of tobacco users who experience some of the most significant health disparities in the US do not have the telephonic resources to use quitlines, our most ubiquitous tobacco treatment modality. These findings also suggest that knowledge about and trust in the quitline is remarkably low among these groups, at least in the Mississippi Delta region. These findings also indicate that there are broad cessation-related factors that contribute to non-use of the quitline including significant concerns about getting sick when one quits and strong faith-based beliefs about cessation. More research is needed into how to best deliver treatment to groups with tobacco disparities who do not have telephonic services that can be used for the quitline and used free of charge, how to provide knowledge about the quitline to those with appropriate telephonic services, and how to address specific concerns about using quitting and other treatment programs. More research is also needed into cessation-related barriers more broadly that prevent use of the quitline and other treatment programs including how to help these and similar groups enhance motivation in difficult circumstances; and perhaps how to incorporate faith-based and other cultural elements that support efforts at quitting. Fully understanding and addressing these barriers might enhance the positive valance of the quitline among those with telephonic resources and treatment programs in general among groups with tobacco disparities as well as increase rates of cessation, and reduce the remarkable disparities in tobacco use prevalence rates.

## References

[1] Centers for Disease Control and Prevention (2011). Current cigarette smoking prevalence among working adults—United States, 2004–2010. MMWR Morb. Mortal. Wkly. Rep..

[2] Centers for Disease Control and Prevention (2011). Vital signs: Current cigarette smoking among adults aged ≥18 years—United States, 2005–2010. MMWR Morb. Mortal. Wkly. Rep..

[3] ACS (2013). Cancer Facts and Figures 2013.

[4] Jha P., Peto R., Zatonski W., Boreham J., Jarvis M.J., Lopez A.D. (2006). Social inequalities in male mortality, and in male mortality from smoking: Indirect estimation from national death rates in England and Wales, Poland, and North America. Lancet.

[5] Mokdad A.H., Marks J.S., Stroup D.F., Gerberding J.L. (2004). Actual causes of death in the United States, 2000. JAMA.

[6] Kanjilal S., Gregg E.W., Cheng Y.J., Zhang P., Nelson D.E., Mensah G., Beckles G.L. (2006). Socioeconomic status and trends in disparities in 4 major risk factors for cardiovascular disease among U.S. adults, 1971–2002. Arch. Intern. Med..

[7] Centers for Disease Control and Prevention (2014). 2014 Surgeon General’s Report: The Health Consequences of Smoking—50 Years of Progress.

[8] Centers for Disease Control and Prevention (2013). Behavioral Risk Factor Surveillance System Survey Data.

[9] Bishaw A., Stern S. (2006). Evaluation of Poverty Estimates: A Comparison of American Community Survey and the Current Population Survey.

[10] U.S. Census Bureau (2000). Census 2000 Urban and Rural Classification. www.census.gov.

[11] CDC (2007). Best Practices for Comprehensive Tobacco Control Programs.

[12] Cummins S.E., Bailey L., Campbell S., Koon-Kirby C., Zhu S.H. (2007). Tobacco cessation quitlines in North America: A descriptive study. Tob. Control.

[13] Lichtenstein E., Zhu S.H., Tedeschi G.J. (2010). Smoking cessation quitlines: An underrecognized intervention success story. Am. Psychol..

[14] Varghese M., Sheffer C.E., Stitzer M., Landes R., Brackman S.L., Munn T. (2014). Socioeconomic disparities in telephone-based treatment of tobacco dependence. Am. J. Public Health.

[15] North American Quitline Consortium Increasing the Reach of Tobacco Cessation Quitlines: A Review of the Literature and Promising Practices. http://www.naquitline.org/resource/resmgr/issue_papers/naqc_issuepaper_increasingre.pdf.

[16] U.S. Census Bureau (2010). US Census Profile of General Demographic Characteristics.

[17] Barnett E., Capser M., Halverson J., Elmes G. (2001). Men and Heart Disease: An Atlas of Racial and Ethnic Disparities in Mortality.

[18] Capser M., Barnett E., Halverson J., Elmes G. (2000). Women and Heart Disease: An Atlas of Racial and Ethnic Disparities in Mortality.

[19] Felix H., Stewart M.K. (2005). Health status in the Mississippi River Delta region. South. Med. J..

[20] Sheffer C. (2007). Arkansas Statewide Tobacco Programs and Services Annual Report 2005–2006.

[21] Lazev A., Vidrine D., Arduino R., Gritz E. (2004). Increasing access to smoking cessation treatment in a low-income, HIV-positive population: The feasibility of using cellular telephones. Nicotine Tob. Res..

[22] Griffin J.L., Segal K.S., Nahvi S. (2015). Barriers to telephone quitline use among methadone-maintained smokers. Nicotine Tob. Res..

[23] Sheffer C.E., Brackman S.L., Cottoms N., Olsen M. (2011). Understanding the barriers to use of free, proactive telephone counseling for tobacco dependence. Qual. Health Res..

[24] Israel B.A., Parker E.A., Rowe Z., Salvatore A., Minkler M., López J., Butz A., Mosley A., Coates L., Lambert G. (2005). Community-based participatory research: Lessons learned from the Centers for Children’s Environmental Health and Disease Prevention Research. Environ. Health Perspect..

[25] U.S. Census Bureau (2008). American Community Survey, Selected Population Profile.

[26] Clinical Practice Guideline (2008). Treating Tobacco Use and Dependence: 2008 Update.

[27] Robertson L., Iosua E., McGee R., Hancox R.J. (2015). Nondaily, low-rate daily, and high-rate daily smoking in young adults: A 17-year follow-up. Nicotine Tob. Res..

[28] Hinkle D.E., Wiersma W., Jurs S.G. (1988). Applied Statitistics for the Behavioral Sciences.

[29] Abdi H. (2007). Bonferroni and Sidak Corrections for Multiple Comparisons.

[30] Glaser B., Strauss A. (1967). The Discovery of Grounded Theory.

[31] Christiansen B., Reeder K., Hill M., Baker T.B., Fiore M.C. (2012). Barriers to effective tobacco-dependence treatment for the very poor. J. Stud. Alcohol Drugs.

[32] Reid J.L., Hammond D., Boudreau C., Fong G.T., Siahpush M. (2010). Socioeconomic disparities in quit intentions, quit attempts, and smoking abstinence among smokers in four western countries: Findings from the International Tobacco Control Four Country Survey. Nicotine Tob. Res..

[33] Kelly D.L., Raley H.G., Lo S., Wright K., Liu F., McMahon R.P., Moolchan E.T., Feldman S., Richardson C.M., Wehring H.J. (2012). Perception of smoking risks and motivation to quit among nontreatment-seeking smokers with and without schizophrenia. Schizophr. Bull..

[34] Karvinen K.H., Carr L.J. (2014). Does the perception that God controls health outcomes matter for health behaviors?. J. Health Psychol..

[35] Shiffman S., Tindle H., Li X., Scholl S., Dunbar M., Mitchell-Miland C. (2012). Characteristics and smoking patterns of intermittent smokers. Exp. Clin. Psychopharmacol..

